# Tracing the lactate shuttle to the mitochondrial reticulum

**DOI:** 10.1038/s12276-022-00802-3

**Published:** 2022-09-08

**Authors:** George A. Brooks, Casey C. Curl, Robert G. Leija, Adam D. Osmond, Justin J. Duong, Jose A. Arevalo

**Affiliations:** Exercise Physiology Laboratory, Department of Integrative Biology, University of California, Berkeley, CA 94720-3140 USA

**Keywords:** Mitochondria, Metabolic syndrome

## Abstract

Isotope tracer infusion studies employing lactate, glucose, glycerol, and fatty acid isotope tracers were central to the deduction and demonstration of the Lactate Shuttle at the whole-body level. In concert with the ability to perform tissue metabolite concentration measurements, as well as determinations of unidirectional and net metabolite exchanges by means of arterial–venous difference (a-v) and blood flow measurements across tissue beds including skeletal muscle, the heart and the brain, lactate shuttling within organs and tissues was made evident. From an extensive body of work on men and women, resting or exercising, before or after endurance training, at sea level or high altitude, we now know that Organ–Organ, Cell–Cell, and Intracellular Lactate Shuttles operate continuously. By means of lactate shuttling, fuel-energy substrates can be exchanged between producer (driver) cells, such as those in skeletal muscle, and consumer (recipient) cells, such as those in the brain, heart, muscle, liver and kidneys. Within tissues, lactate can be exchanged between white and red fibers within a muscle bed and between astrocytes and neurons in the brain. Within cells, lactate can be exchanged between the cytosol and mitochondria and between the cytosol and peroxisomes. Lactate shuttling between driver and recipient cells depends on concentration gradients created by the mitochondrial respiratory apparatus in recipient cells for oxidative disposal of lactate.

## Metabolism: Reviewing the lactate shuttle

Studies using isotope tracer technologies have significantly improved understanding of how lactate, a metabolite produced as fuel during normal metabolism and in response to exercise, moves or ‘shuttles’ throughout the body. George Brooks and colleagues at the University of California, Berkeley, USA, reviewed the history of the understanding of lactate shuttling, which has largely been informed by human studies using isotope tracer infusions during rest and exercise. Such research highlights continuous organ–organ, cell–cell, and intracellular lactate shuttling. Lactate moves between producer cells such as skeletal muscle cells and consumer cells in tissues including the heart and brain, where it is preferred over glucose as an energy source. Shuttling depends on lactate concentration gradients created by mitochondrial networks in recipient cells. Lactate is disposed of via oxidation.

## Introduction

The results of isotope tracer studies at the whole-body and tissue levels have clarified longstanding issues concerning metabolic regulation. In particular, the role of the mitochondrial reticulum^1,2^ in lactate disposal has been identified. The reticulum lowers cellular [lactate] by oxidative disposal, thus acting to develop the concentration gradients necessary for lactate fluxes across organ, tissue, and cellular compartments. Similar to other solutes, gases, and entities in physics and physiology, lactate moves from areas of high to areas of low concentrations. Hence, lactate-producing cells, organs, and tissues with high lactate levels drive carbon flux to disposal (recipient) sites with low lactate levels. In this scenario, the mitochondrial reticulum, in which lactate is disposed of via oxidation, creates the driving force for shuttling lactate around the body and within organs, tissues and cells^3–5^. Key features of the process involve the mitochondrial (m) Lactate Oxidation Complex (mLOC), which contains mitochondrial lactate dehydrogenase (mLDH), a monocarboxylate (lactate) transporter (mMCT), a scaffolding protein (CD147) and cytochrome oxidase (COx). However, within the context of this special issue on the use of isotopic tracers to study metabolism, the results of lactate tracer studies directly led to the discovery of the mLOC for lactate shuttling to occur in vivo. We begin with a discussion of mLDH.

Similar to the concept of an mLOC, discussions on the role of mLDH in intermediary metabolism have endured a long and winding history^3,5,6^, perhaps a path not unusual in the history of science^3,7,8^. Because of the fundamental importance of mLDH in physiology and metabolism, revisiting the history of discoveries is warranted at the outset of this paper. The presence of mLDH is essential to explain the role of lactate in affecting lactate flux and ultimately the regulation of energy substrate partitioning in vivo^3,5,9^.

### Background knowledge

Today, we know that some types of facultative cells can be cultured with lactate as a preferred fuel because their mitochondrial reticulae consume and oxidize lactate directly without conversion to pyruvate in the cytosol. For instance, mitochondria of yeast (*Saccharomyces cerevisiae*) contain Flavocytochrome *b*_2_, a lactate-cytochrome c oxidoreductase^10^ that couples lactate dehydrogenation to the reduction of cytochrome c^11^. In fact, the association between cytochrome *b*_2_ and LDH in yeast can be traced to the 1940s^12^. A similar phenomenon occurs in mammalian muscle^13–19^, in persons resting at the sea level, and even in men exercising at sea level or at an altitude of 4300 m^20,21^. Technological limitations, such as the availability of isotope tracer methodology, the ability to catheterize veins and arteries for continual (a-v) measurements, the ability to determine tissue and systemic blood flow rates, and mass spectrometry for turnover analyses, made such data unobtainable in the 1920s. Consequently, longstanding ideas on the role of lactate in physiology originated on the basis of incomplete data on isolated, nonperfused, nonoxygenated amphibian muscles made to contract continually in ways not intended by nature^22–27^. More recently, with the advent of isotope tracers and nuclear magnetic resonance (NMR) spectroscopy, we now know that contractions stimulate glycolysis in muscle independent of O_2_ availability^28–33^ and that lactate is oxidized in working skeletal^31,34,35^ and cardiac^33,36–39^ muscles.

## Mitochondrial lactate oxidation (the mind’s eye view of Polyphemus)

Contemporary textbooks of biochemistry and physiology abound the 19th-century concepts that metabolism is either “anaerobic” (without O_2_) or “aerobic” (with O_2_)^40^. Glycolytic flux from glucose and glycogen is typically depicted in textbooks as progressing to pyruvate and then to the tricarboxylic acid (TCA) cycle. However, if oxygen is absent, textbooks assert that glycolysis progresses to lactate^41–43^. This is a convenient motif, typically copied by one textbook author from another and then through serial editions of texts. Amazingly, some textbook authors who advanced the idea of lactate production due to oxygen limitation were biochemists who worked with cells in high-glucose-containing culture media under fully aerobic conditions of one atmosphere pressure in which the partial pressure of oxygen was at least 50% greater than in the arterial blood of individuals at sea-level altitudes. Because the maintenance of cells in such preparations required daily changing of the incubation media to maintain high [glucose], low [lactate], and physiological pH, the observations should have informed the investigators that lactate was produced under fully aerobic conditions. Contemporaneously, physiologists measured lactate [L] to pyruvate [P] concentration ratios (L/P) of 10 in muscles and blood in resting mammals, including humans, and further observed the L/P to rise more than 100 during submaximal exercise^44^. However, did any of the textbook authors ever look to determine whether isolated mitochondria oxidize lactate? For many textbook authors^41–43^, the answer is “no.” However, for a few others, the answer is “yes”^45,46^. Moreover, some investigators also looked for the presence of a mitochondrial monocarboxylate (lactate) transporter (mMCT) and a lactate dehydrogenase (mLDH) enzyme. Unfortunately, while some attempts failed^47–49^, fortunately, other attempts to observe mitochondrial lactate oxidation and the presence of mLDH and mMCT were successful^13,50–55^. Importantly, lactate oxidation in human muscle mitochondrial preparations was observed^56^. Regrettably, for a time, positive results were overlooked or castigated as being “controversial” and dismissed for failing to fit with established paradigms of metabolic regulation. However, now that the MitoCarta (https://www.broadinstitute.org/mitocarta/mitocarta30-inventory-mammalian-mitochondrial-proteins-and-pathways)^57^ and MitoMiner (https://mitominer.mrc-mbu.cam.ac.uk/release-4.0/begin.do) databases have been published, mLDH is a recognized constituent of the mitochondrial proteome. Acknowledging that lactate, not pyruvate, is the main gluconeogenic precursor and carbohydrate energy source shared at the organismal and tissue levels upsets archaic concepts of the organization of intermediary metabolism. Nevertheless, despite the evidence, in the manner of Polyphemus, some^58,59^ have ignored the literature and databases on mLDH and mMCT and their roles in mitochondrial lactate oxidation^60^.

### The lactate:pyruvate affair

Regrettably and inexplicably, thus far, as the use of isotope tracers to study lactate metabolism is concerned, the literature has been muddled, creating a “Lactate:Pyruvate Affair” that needed to be corrected^60^. Specifically, there has been controversy over whether lactate tracers measure lactate (L) or pyruvate (P) turnover. Despite a series of analytical errors, the use of inappropriate tissue and animal models, a failure to consider L and P pool sizes in modeling results, inappropriate tracer and blood sampling sites, a failure to anticipate roles of heart and lung parenchyma on L ⇔ P interactions, and a failure to appreciate results from magnetic resonance spectroscopy (MRS) and immunocytochemistry^61,62^, it is clear that carbon-labeled lactate tracers can be used to quantitate lactate fluxes in situ and in vivo.

### The early twentieth century

In the early literature of the 20th century, there were several threads to a contemporary understanding of lactate production and oxidation during postprandial and exercise conditions. Among those threads were the findings of Fletcher and Hopkins, who showed that lactic acid disappeared when frog muscles fatigued by electrical stimulation were placed in oxygen-rich environments^63^. Albeit in another field of endeavor, cancer research, another thread was the finding of Warburg that lactate was produced by fully oxygenated cancer cells^64^. Nonetheless, while there were data from a reputable investigator (Nobel Laureate) that lactate could be produced under fully aerobic conditions and that oxidative removal of lactate could be accomplished without reconversion to glucose, lactate and lactic acid in cell metabolism were misconstrued as waste products of oxygen-limited metabolism and fatigue agents early in the 20th century^65–67^.

Most importantly, several sets of findings in the late 20th century led to the realization of oxidative lactate disposal. Among these sets of findings was evidence that isolated mitochondrial preparations could oxidize lactate in vitro^13,54^ and that mitochondria contained the enzymatic apparatus to oxidize lactate^13,53,68^. Importantly, isotope tracer studies showed oxidative lactate disposal in mammals and humans in vivo^69–73^. Evidence obtained on individuals studied in vivo is elegantly supported by the results of studies using MRS technology^28,33,34,74^. These important sets of findings are addressed sequentially.

## Overlooked findings

The first investigator to evaluate mitochondrial lactate metabolism was Mario Umberto Dianzani (1925–2014), who, in 1951, published a paper, translation of which is “Distribution of lactic acid oxidase in liver and kidney cells of normal rats and rats with fatty degeneration of the liver”^55^. A translation of a section on p. 182 is “In conclusion, the [cellular] lactic acid-oxidizing systems are localized 80 to 100% in the mitochondria.” Although Dianzani had a prolific career in hepatic toxicology and only recently retired from the Department of Experimental Medicine and Oncology, University of Turin, he apparently never followed up on his finding of mitochondrial lactate oxidation, and his seminal findings went unrecognized even by workers in the field of mitochondrial energetics.

**Fig. 1:**
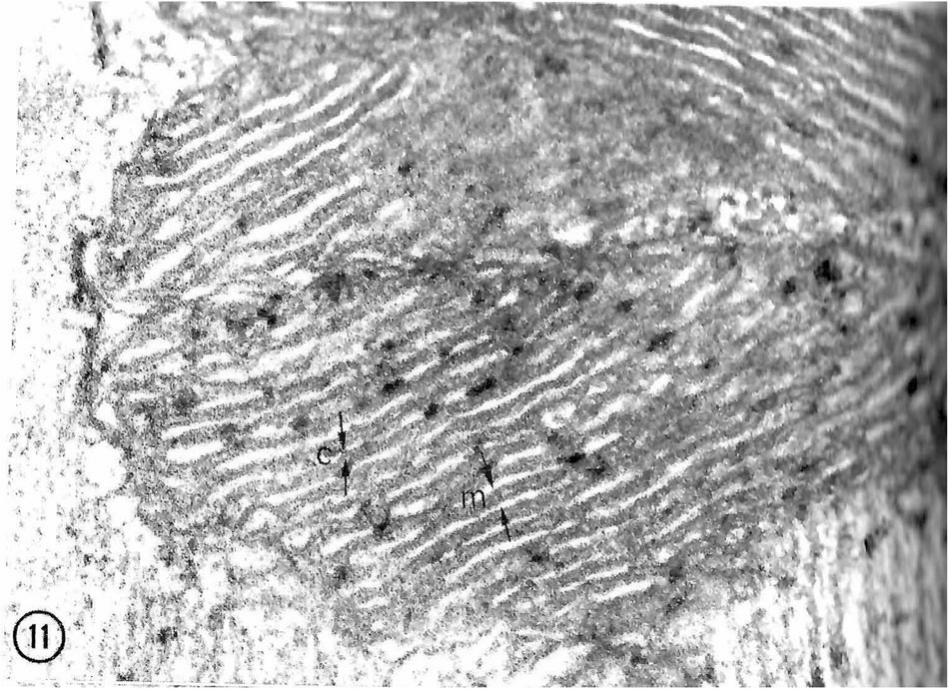
First report of LDH in the myocardium of rat heart. Reaction products of LDH seen in the mitochondria are primarily located within the inner mitochondrial membranes (C, cristae). M matrix. X 93,000. Figure 11 from Baba and Sharma^53^.

**Fig. 2:**
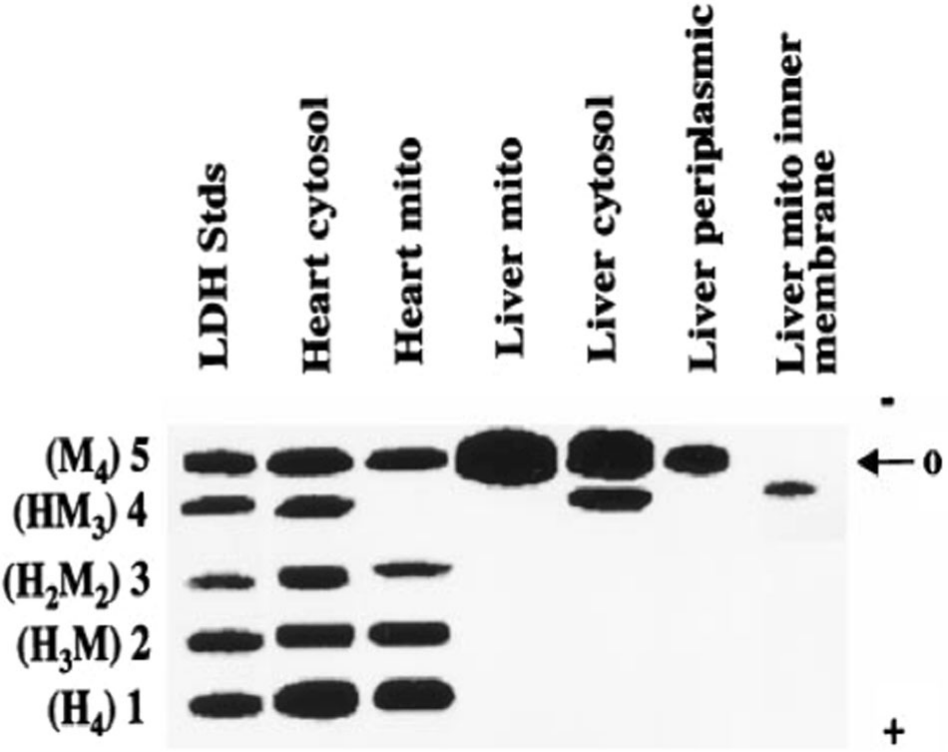
First report using agarose gel electrophoresis to separate LDH isoforms in mitochondria from rat liver and heart. LDH isoenzyme patterns differ between the cytosol and mitochondria in both tissues. Note cytosolic and mitochondrial LDH isoform differences between the cytosol and mitochondria across tissues. From Brooks et al.^13^.

First to report the presence of mitochondrial LDH were Nobuhisa Baba and Hari M. Sharma, who used electron microscopy (EM) to study the hearts and pectoralis muscles of rats^53^ (Fig. 1). Decades later, their results showing mLDH were confirmed by many others, including Brandt and Kline et al.^51,52,75^, Brooks et al.^13^, Nakae et al.^76^, Taylor et al.^77^, Lemire^16^, Szczesna-Kaczmarek et al.^18,19^, Young et al.^54^, and Pagliarini et al.^57^. Subsequently, the results were extended to human skeletal muscle mitochondria by Dubouchaud et al. in the Brooks Laboratory^15^ (Fig. 2). Retrospectively, based on their seminal observations of mLDH using EM, Baba and Sharma were probably the first to use the term “lactate shuttle.” Regrettably, they never followed up on their original finding of mLDH, and similar to the discovery of Dianzani, the seminal findings of Baba and Sharma went unrecognized.

Beyond Dianzani^78^ and those who replicated his seminal observation that mitochondria were capable of oxidizing lactate^13,51,52,54,56,79^ were the efforts of those who failed in their attempts to isolate mitochondrial preparations that contained sufficient mLDH to oxidize lactate^47–49,80,81^. In addition, there were others who routinely found LDH in their mitochondrial preparations but regarded their findings as an artifact and took steps to block mLDH, thus permitting the measured rate of exogenous pyruvate oxidation to rise^82^. The lesson to take from history is that mLDH is necessary for lactate oxidation. When mLDH is lost in the isolation of single fibers^49^ or mitochondrial vesicles^68^ or if oxamate is used to block mLDH^13^, the preparation will be able to oxidize pyruvate but not lactate.

The ability of the mitochondrial reticulum to respire lactate is fundamental to the operation of lactate shuttles because lactate disposal via oxidation in the reticulum decreases the cellular [lactate], thus establishing lactate concentration gradients down which lactate fluxes. Hence, it is understandable that the rates and directions of lactate flux vary with physiological conditions (e.g., rest or exercise, early or late during exercise, endurance-trained or sedentary individuals, sea level or high altitude, carbohydrate nourished). For instance, in the postprandial state, when red skeletal muscles are glucose consumers and lactate producers^4^, and hence drivers of lactate shuttling, the heart, liver, and kidneys are consumers or recipients of lactate shuttle. In contrast, during exercise, white skeletal muscle and the integument are lactate producers and drivers of lactate shuttling, whereas highly oxidative (red) fiber types, cardiac tissue, liver, kidneys, and brain^83–87^ are sites of net lactate disposal. In these and other forms of shuttling, lactate fluxes from high to low concentrations with the cellular respiratory (mitochondrial) apparatus, including mLDH, functioning as removal sites^3,5,88,89^. These phenomena underpin the tracer-measured flux rates observed under conditions of rest and exercise^35,90,91^.

In a previous publication, the presence of a Postprandial Lactate Shuttle was introduced. Following a carbohydrate (CHO) meal, glycolysis, and lactate production in noncontracting red skeletal muscle^92,93^ raises lactate in the systemic circulation, thus providing a substrate for hepatic and renal gluconeogenesis^85,94^ and an energy substrate for skeletal muscle^35,91,95^ and the heart^39,95,96^, thereby forming a Postprandial Lactate Shuttle^4^.

## Tracers and lactate turnover during rest and exercise

Pioneering work in the field of lactate kinetics in exercising mammals was performed by Florent Depocas (1923–2004) and colleagues^69^. Using a continuous infusion of [U-^14^C]lactate into dogs during rest and continuous steady-state exercise, they made several key, fundamental findings regarding lactate metabolism. These findings included (1) active lactate turnover during the resting postabsorptive condition; (2) ~1/2 of lactate formed during rest is removed through oxidation; (3) the turnover rate of lactate increases during exercise compared to rest even if there is only a minor change in blood lactate concentration; (4) the fraction of lactate disposal through oxidation increases to approximately 3/4 during exercise, and (5) a minor fraction (1/10-1/4) of lactate removed is converted to glucose via the Cori Cycle during exercise. Although the fractions are subject to species and experimental variations, the essential results have been reproduced in rats^71^, rabbits^97^, dogs^69,98,99^, horses^100^, and humans^21,35,36,70,73,91,101^. Depocas’ work on lactate metabolism was just one small part of his work at the Canadian NRC, where he held wide-ranging interests in the metabolic and endocrine responses of mammals to hypothermia.

Over the span of two decades, commencing in the mid-1980s, Brooks Lab Staff Research Associates Michael A. Horning, Gretchen C. Casazza and Jill A. Fattor; Brooks’ graduate students Timothy P. White, Glenn A. Gaesser, Casey M. Donovan, William C. (Bill) Stanley, Robert S. Mazzeo, Bryan C. Bergman, Benjamin F. Miller, David A. Roth, Sang-Hoon Suh, Rajaa Hussien, and Chi-An W. Emhoff; and postdoctoral fellows, visiting scholars and collaborators such as Thomas D. Fahey, Eugene E. Wolfel, Jacques Mercier, Hashimoto, Hervé Dubouchaud, Grant McClelland and Laurent A. Messonnier made concerted efforts to describe the effect of exercise intensity and training state on whole-body and working muscle lactate oxidation and gluconeogenesis from lactate in humans. The effects of exercise intensity on whole-body lactate turnover and oxidation were initially reported by Stanley^70,91,95^ and Mazzeo^73^, who showed that tracer-measured lactate turnover and oxidation scaled to the metabolic rate during exercise and that while oxidation accounted for ~50% of lactate disposal in resting men, oxidation accounted for 75–80% of lactate disposal during continuous hard exercise.

Studies of lactate metabolism in working skeletal and cardiac muscle in men during exercise were reported by Stanley et al. in 1986^70,91^ and Edward W. Gertz et al. in 1988^39,96^, respectively. The advantage of using tracer infusions along with simultaneous a-v difference and blood flow measurements across working muscle beds is that lactate uptake and net release, therefore, total production (= the sum of uptake and net release), can be determined simultaneously. Furthermore, another advantage of using tracer infusions along with simultaneous a-v ^13^CO_2_ and blood flow measurements across tissue muscle beds is that tissue metabolite oxidation can be measured. In addition, working muscle turnover and oxidation rates can be compared with whole-body rates. The results showed simultaneous lactate extraction (uptake) and release (production) and oxidation by working human leg muscles, which explains most whole-body lactate turnover and oxidation rates^91^. Importantly, due to intramuscular lactate extraction, turnover and oxidation, net chemical balance measurements underestimate true lactate production and oxidation rates for metabolites such as lactate and free fatty acids that turnover within tissue beds^102^. Subsequently, using variations of the same methodology, cerebral glucose, and lactate uptake and release were determined in traumatic brain injury patients and healthy controls^103,104^.

Another relevant but underappreciated and often unmentioned aspect of the issue of blood lactate accumulation during exercise is the assumption that the circulating lactate level rises because of net release from active muscle. Surprisingly, while net lactate release from resting muscle is common, net release from working muscle is usually transient if the power output is submaximal and held constant. As shown first by Wendell Stainsby and Hugh Welch in 1966–1967^105,106^ using dog muscle preparations contracting in situ, this “Stainsby Effect” of transient muscle net lactate release at exercise onset followed by a switch to net uptake from the blood by working muscle has been confirmed in exercising humans^35^. In this regard, noteworthy are the studies of L. Bruce Gladden on dog muscles contracting in situ^107^. Gladden clearly showed that lactate uptake is the concentration (substrate) and not O_2_-dependent, a finding that also appears to be the case in human muscle^35^, vide infra. Thus, it is certain that working skeletal muscle is not the sole source of blood lactate in humans during whole-body exercise. Epinephrine is more likely to signal glycolysis and lactate production in noncontracting tissues than in working muscle; in working muscle, epinephrine augments glycolysis, leading to increased lactate accumulation^108–110^.

### Choice of lactate carbon tracers to determine lactate flux and disposal in vivo

As noted above, initial studies of lactate metabolism in vivo utilized a uniformly labeled tracer, i.e., U-^14^C]lactate^69^. However, with the advent of stable, nonradioactive ^13^C-labeled tracers, it was ethically feasible to study flux and oxidation in human subjects^111^. Because lactate disposal via oxidation gave rise to ^13^CO_2_, carbon-labeled lactate tracers were preferred over heavy hydrogen (i.e., deuterated, D) tracers because the oxidation product (^13^CO_2_) could be detected at the tissue level in the venous effluent of tissues such as muscle^35^, heart^36,39^, and brain^103^ and at the whole-body level by excretion in expired air^35,73,95,112^. Another advantage of the ^13^C-lactate tracer was that it could be used in combination with a deuterated glucose tracer, e.g., [6,6-^2^H]glucose, a so-called “irreversible tracer”, because the D atoms are lost to body water during glycolysis. Moreover, because a percentage (≈25%) of ^13^C atoms from lactate are reincorporated into glucose during the process of gluconeogenesis, the combination of [3-^13^C]lactate and [6,6-^2^H]glucose (i.e., D2-glucose) could be used to simultaneously study lactate and glucose flux rates, the lactate oxidation rate and the rate of gluconeogenesis from lactate^113–115^.

On the issue of which isotope tracer of lactate to use, the most common is [3-^13^C]lactate, which yields information about total lactate disposal as well as the components of oxidation and gluconeogenesis. Earlier use of uniformly labeled compounds to determine metabolite disposal rates was abandoned because of the extensive recycling and exchange of labeled lactate carbon atoms^116,117^. Of the alternatives, ^13^C from [1-^13^C]lactate is lost as ^13^CO_2_ in the pyruvate dehydrogenase reaction, as pyruvate is converted to acetyl-CoA upon entry into the TCA (Krebs) Cycle. This decarboxylation step shows entry into the TCA cycle, but perhaps not complete oxidation of the lactate molecule. For that, [3-^13^C]lactate is the preferred tracer to demonstrate lactate oxidation downstream in the TCA cycle by the actions of isocitrate and alpha-ketoglutaric dehydrogenases^41,118^.

Finally, with the use of carbon-labeled tracers to determine the oxidative disposal of lactate, it needs to be acknowledged that because metabolically produced ^13^CO_2_ enters the body’s bicarbonate-CO_2_ pool, a separate experiment under identical conditions needs to be conducted. At rest, the bicarbonate-CO_2_ pool turns over slowly, so for the short term (1 to several hours), an isotope dilution factor needs to be applied^119^. In resting humans, the correction factor for CO_2_ retention is large (≈50%)^120^. The large correction factor for estimating isotopic dilution and retention is daunting for investigators to make because it is so large. Fortunately, however, during exercise, the necessity of a bicarbonate correction factor disappears because the bicarbonate-CO_2_ pool turns over rapidly with no measurable ^13^CO_2_ retention or diversion of tracer to other, unmeasured metabolite pools^121,122^. In addition, some investigators have explored the use of so-called “acetate correction factors”^123^. However, the latter approach is not recommended because acetate is not bicarbonate and, most importantly, because the C1 and C2 of acetate behave differently, leaving an investigator uncertain about what the appropriate correction factor is^120^.

## The lactate shuttle and membrane lactate transport and lactate/pyruvate exchange

With knowledge of glucose and lactate fluxes and oxidation rates in resting and exercising rats^71,124^, in 1984, George A. Brooks took a unique approach to explain phenomena related to lactate responses to exercise when the “Lactate Shuttle Hypothesis” was articulated^125^. A key element of the hypothesis was that “the shuttling of lactate through the interstitium and vasculature provides a significant carbon source for oxidation and gluconeogenesis during rest and exercise”. As such, the lactate shuttle hypothesis represents a model of how the formation and distribution of lactate represents a central means by which the coordination of intermediary metabolism in diverse tissues and different cells within tissues can be accomplished. The initial hypothesis was developed from the results of original isotope tracer studies conducted on laboratory rats in the Brooks Laboratory along with numerous other studies, many of which were cited in the previous section. Thus, the working hypothesis was developed that much of the glycolytic flux during exercise passed through lactate.

According to the Cell–Cell Lactate Shuttle hypothesis, lactate is a metabolic intermediate rather than an end product^3,88,126–129^. Lactate is continuously formed in and released from diverse tissues, such as skeletal muscle, skin, and erythrocytes, and also serves as an energy source in highly oxidative tissues, such as the heart, and is a gluconeogenic precursor for the liver and kidneys. Lactate exchanges among these tissues appear to occur under various conditions, ranging from postprandial rest to sustained postabsorptive exercise^88,89,125^.

If lactate does serve as a key metabolic intermediate that shuttles into and out of tissues at high rates, particularly during exercise, then transmembrane movement is critical. For many years, lactate was assumed to move across membranes by simple diffusion. However, by 1980, facilitated protein carrier-mediated transport of lactate across erythrocyte membranes was well documented^130,131^. It was not until 1990 that the characteristics of sarcolemmal membrane lactate transport were described^132–134^. The study of cell membrane lactate transport proteins took another major leap in 1994. When looking for the mevalonate (Mev) transporter gene in Chinese hamster ovary cells, Christine Kim Garcia in the Goldstein and Brown laboratories cloned and sequenced a monocarboxylate transporter that she and her colleagues termed the monocarboxylate transporter (MCT)^135^. In 1985, Goldstein and Brown shared the Nobel Prize for “their discoveries concerning the regulation of cholesterol metabolism”^136^. The newly discovered MCT was abundant in erythrocytes, the heart, and the basolateral intestinal epithelium. MCT was also detectable in oxidative muscle fiber types but not in the liver. With an interest in describing a role for MCT isoforms in the Cori Cycle, Garcia et al.^137^ subsequently described the isolation of a second isoform (MCT2, in effect renaming the first transporter discovered to MCT1) by screening a Syrian hamster liver library; MCT2 was initially found in the liver and testes but subsequently also in the brain^138,139^ and some tumor cell lines^16,140,141^.

Independent of Garcia et al.^135,137^, Price et al.^142,143^ identified another MCT isoform (now known as MCT4) in 1998. In terms of physiology, Halestrap and colleagues^144^ continued to hold traditional, Hill-Meyerhof views of an association between lactate transporter expression and oxidative metabolism even though their data showed a high correlation between MCT1 expression and mitochondrial markers^144,145^, which was subsequently replicated by Dubouchaud et al.^15^. However, with a different concept based on the knowledge that lactate was formed and oxidized continuously within muscle and heart tissue in vivo, Brooks and associates sought to identify the molecular basis of the coupling of glycolytic with oxidative pathways. In that pursuit, Brooks and colleagues first observed the presence of LDH in rat liver, cardiac and skeletal muscle mitochondria^15^. Subsequently, they also showed MCT1 to be in mitochondria of the same tissues^50^, thus allowing mitochondria to directly oxidize lactate^13^. With such knowledge, in 1998, Brooks extended the Cell–Cell Lactate Shuttle concept to include an intracellular component, the “Intracellular Lactate Shuttle.” As already noted, several of the observations, such as the presence of mLDH^53^ and mitochondrial lactate oxidation^55^, were similar to the vision of Polyphemus—without depth perception, swamped by the mediocrity of authority, buried and forgotten in the literature for a time but later resurrected^3,4^.

## Compartmentation issues and controversies—the intracellular lactate shuttle

While there is growing agreement on some aspects of the Cell–Cell Lactate Shuttle, within exercise physiology and other fields, such as neurobiology^139,146–150^ and cancer research^140,141,151,152^, some aspects of the Intracellular Lactate Shuttle remain controversial. In essence, disagreement exists over where the first step in lactate oxidation (i.e., conversion to pyruvate) occurs. This is why a discussion over whether mitochondria contain LDH persists. If so, identifying where LDH would reside so that it is able to oxidize lactate (vide supra) is important. However, the results of light and electron microscopy studies conducted to date show that mLDH resides within the mitochondrial reticulum.

### Electron microscopy

Prior to the MitoCarta and MitoMiner databases based on proteomics, those who looked for muscle mLDH located it in tissues. Studying rat heart, Baba and Sharma used methods to protect mLDH during tissue fixation for electron microscopy. Reaction products of mLDH seen in the mitochondria were “primarily located within the cristae” (Fig. 1) (their Fig. 11)^53^. In addition, they reported similar results for rat pectoralis Types I and II fibers. Similarly, using an antibody to LDH and gold particle secondary labeling, Brooks et al. also showed LDH located on the inner mitochondrial membranes of rat muscle, heart, and liver^13^. Moreover, they used agarose gel electrophoresis to separate LDH isoforms in the cytosol and mitochondria isolated from rat livers and hearts. LDH isoenzyme patterns differed between the cytosol and mitochondria in both tissues (Fig. 2).

### Immunocytochemistry

**Fig. 3:**
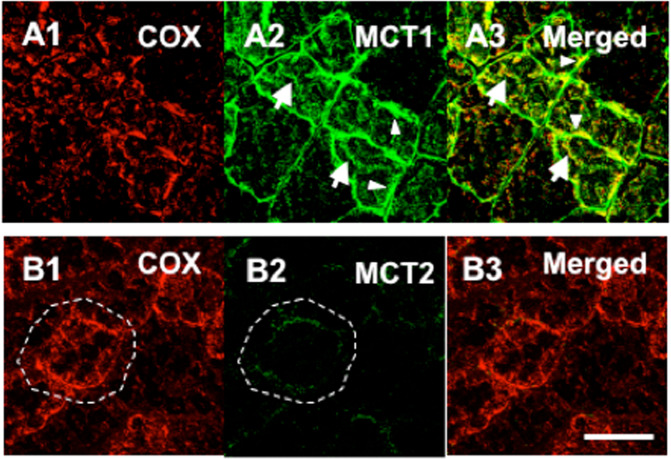
Plasma and mitochondrial membrane locations of monocarboxylate transporters (MCTs). Figures showing the cellular locations of MCT1 and MCT2 lactate transporter isoforms and the mitochondrial reticulum (cytochrome oxidase, COx) in adult rat plantaris muscle determined using confocal laser scanning microscopy (CLSM) and fluorescent probes for the respective proteins; comparisons for MCT1 in the first row (Plates **A1**–**A3**, respectively) and for MCT2 in the second row (Plates **B1**–**B3**, respectively). The localization of COx was detected in rat plantaris muscle (Plates **A1** and **B1**). MCT1 was detected throughout the cells, including the subsarcolemmal (arrowheads) and interfibrillar (arrows) domains (Plate **A2**). MCT1 abundance was greatest in oxidative fibers where COx was abundant and the signal was strong. When signals from probes for MCT1 (green) and COx (red) were merged, the superimposition of the two probes was clear (yellow), a finding prominent at the interfibrillar (arrows) and sarcolemmal (arrowheads) cell domains (Plate **A3**). In contrast, the signal for MCT2 (Plate **B2**) was weak and relatively more noticeable in fibers denoted by strong staining for COx (Plates **B1** and **B3**, broken line delineated around oxidative fibers to distinguish the faint signal for MCT2). The overlap of MCT2 and COx is insignificant, denoted by the absence of yellow in Plate B3. Scale bar = 50 μm. Sections are from the same animal. Reprinted from Hashimoto et al.^154^.

**Fig. 4:**
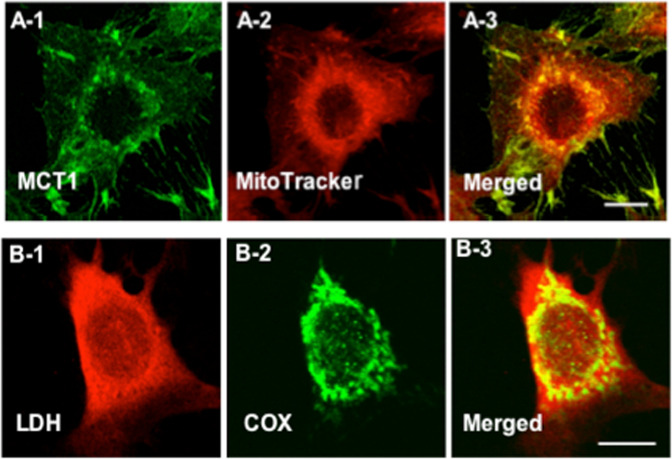
Immunohistochemical images demonstrating some components of the Mitochondrial Lactate Oxidation Complex (mLOC) in cultured L6 muscle cells. This complex involves the mitochondrial constituent cytochrome oxidase (COx), lactate-pyruvate transport protein (MCT1), lactate dehydrogenase (LDH), and other constituents. **A** Colocalization of MCT1 and the mitochondrial reticulum. MCT1 was detected at both sarcolemmal and intracellular domains (**A-1**). Using MitoTracker, the mitochondrial reticulum was extensively elaborated and detected at intracellular domains throughout L6 cells (**A-2**). When signals from probes for the lactate transporter (MCT1, green, **A-1**) and mitochondria (red, **A-2**) were merged, the superimposition of the signals (yellow) showed the colocalization of MCT1 and components of the mitochondrial reticulum, particularly at perinuclear cell domains (**A-3**). In Panel (**B**), lactate dehydrogenase (LDH) (**B-1**), and mitochondrial cytochrome oxidase (COx) (**B-2**) are imaged. The superimposition of signals for LDH (red, **B-1**) and COx (green, **B-2**) shows the colocalization of LDH in the mitochondrial reticulum (yellow) of cultured L6 rat muscle cells (**D-3**). Depth of field ~1 μm, scale bar = 10 μm. Reprinted from Hashimoto et al.^153^.

**Fig. 5:**
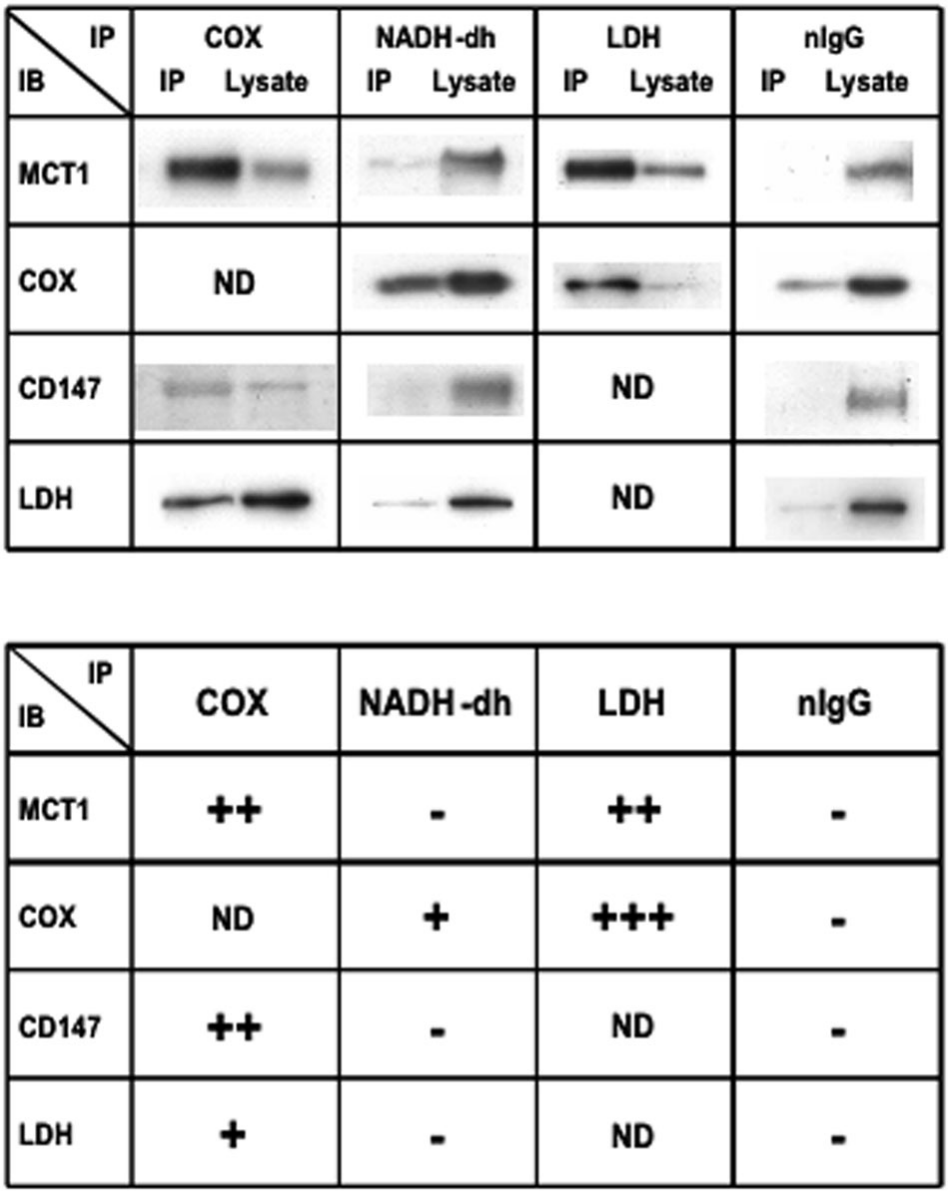
Results of efforts to deduce the organization of the mLOC using immunoprecipitation (IP) technology. In the upper panel, representative immunoblots (IB) are shown using anti-COX, NADH-dh, LDH, or nIgG as precipitating antibodies (IPs). COX, NADH-DH, LDH, and nIgG were immunoprecipitated from mitochondrial fractions of L6 cells resuspended in a suspension medium without detergent. COX IP proteins were probed with MCT1, CD147, and LDH antibodies. MCT1, CD147, and LDH were coprecipitated with COX. NADH-dh IP pellets were probed with MCT1, COX, CD147, and LDH antibodies. Neither MCT1, CD147, nor LDH coprecipitated with NADH-DH, whereas COX coprecipitated with anti-NADH-dh. LDH IP proteins were probed with MCT1 and COX antibodies. Both MCT1 and COX coprecipitated with LDH. No protein coprecipitated with nIgG from mitochondrial fractions of L6 cells resuspended in medium without detergent (negative control). In the lower panel, the degree of coprecipitation evaluated by comparing signals in the IP and the lysate is shown. The results were categorized into four levels: +++, 80% or more precipitated; ++, ∼50% precipitated; +, 20% or less precipitated; -, no precipitation. IB immunoblot, IP immunoprecipitation. From Hashimoto et al.^138^.

**Fig. 6:**
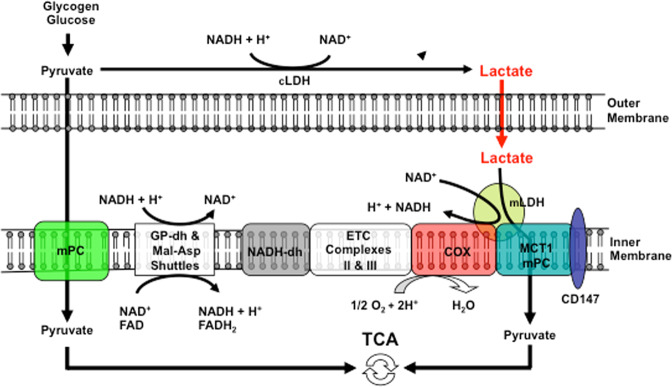
Schematic showing the putative lactate oxidation complex. Lactate is oxidized to pyruvate via mitochondrial LDH (mLDH) in association with COx. This endergonic lactate oxidation reaction is coupled to the exergonic redox change in COx during mitochondrial electron transport. The transport of pyruvate across the inner mitochondrial membrane is facilitated by MCT1. GP glycerol phosphate, Mal-Asp malate-aspartate, ETC electron transport chain, TCA tricarboxylic acid. Figures 4 and 5 show the results of mitochondrial respiration studies^13,50,56^. Modified from Hashimoto et al.^138^.

In the Brooks Laboratory, Takeshi Hashimoto used confocal laser scanning microscopy, immunoprecipitation (IP), and cell fractionation techniques followed by western blotting to verify the presence of the purported Mitochondrial Lactate Oxidation Complex (mLOC) in cultured myocytes as well as in thin sections of adult rat tissues. The colocalization of MCT1, mitochondrial cytochrome oxidase (COx) and LDH in the mitochondria of adult rat skeletal muscle and astrocytes was visualized by means of combinations of primary and fluorescently labeled secondary antibodies plus MitoTracker Red and dual-wavelength scanning confocal microscopy (Fig. 3)^138,153,154^. Subsequently, similar techniques allowed the investigators to show the colocalization of mLOC components in cultured L6 (rat muscle-derived) cells (Fig. 4). Again, those results were confirmed by two independent techniques—IP and Western blotting of isolated cell fractions^138,153–155^. The results of IP efforts are shown in Fig. 5, and a pictorial representation of how the mLOC is organized on the inner mitochondrial membrane with projection into the intermembrane space is constructed from the results of immunocytochemistry and immunoprecipitation studies (Fig. 6).

Both morphometry and the susceptibility to loss during mitochondrial isolation suggest that LDH resides in the intermembrane space or, similar to cytochrome c, is loosely fixed to the inner membrane. On the other hand, if it is acknowledged that cardiac and red skeletal muscle take up and oxidize lactate, while the presence of mLDH and an mLOC are denied, then opponents of the Intracellular Lactate Oxidation Complex must rely on cytosolic oxidation of lactate to pyruvate. Cytosolic oxidation of lactate is unlikely^156^ and contrary to the evidence available from Peter Hochachka and colleagues, who studied glycolysis in a variety of mammalian species^157,158^, and Bradley Zinker et al.^159^ in the David Wasserman laboratory, who studied glucose and lactate metabolism in the hind limb muscles of running dogs. By means of tracer glucose, they showed intramuscular lactate production from glucose to occur simultaneously with net muscle lactate uptake^159^. Clearly, glycolysis progressed to lactate production in the working muscles of running dogs, whereas lactate oxidation occurred in a compartment where it was oxidized, the mitochondrial reticulum.

### Mitochondrial monocarboxylate uptake mMCTs, mPCs or Both?

**Fig. 7:**
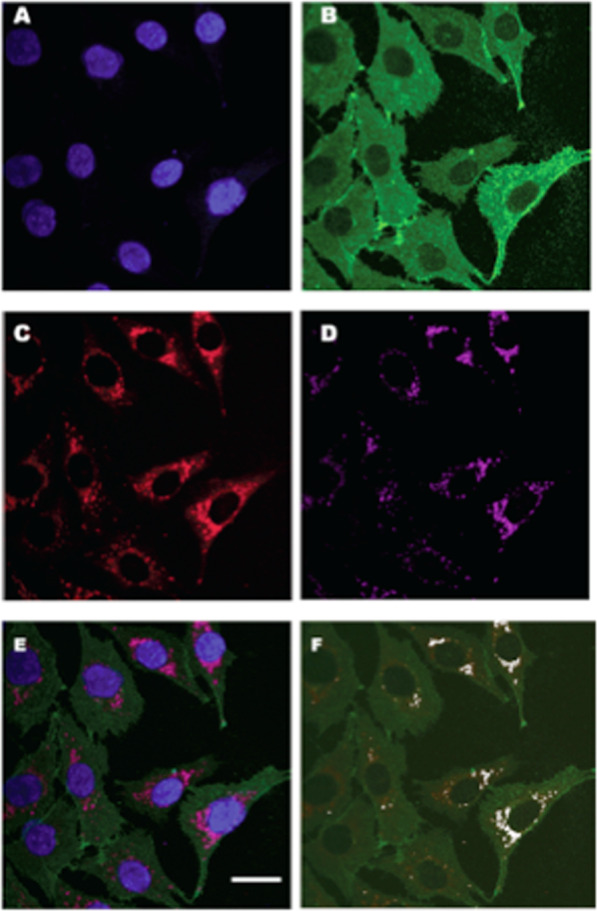
Cellular colocalization of mitochpondrial lactate (VCTs) and pyruvate transprters (mPCs). Images assessing the colocalization of MCT1 and mPCs in L6 cells, which show the localization of DAPI-positive nuclei (**A**), MCT1 (**B**), mPC1 (**C**), and MitoTracker-positive MR (**D**) in L6 cells. The merged images are shown in (**E**). Colocalization analysis of mPC1 (**C**) and mitochondria **D** showed a Pearson correlation coefficient (*r*^2^) value of 0.8. Colocalization analysis of MCT1 (**B**) and mPC1 (**C**) showed an *r*^2^ of 0.3, largely because MCT1 occupies sarcolemmal, mitochondrial and peroxisomal compartments. A channel to represent the colocalization of MCT1 and mitochondria was created to image mMCT1; subsequent colocalization of mMCT1 with mPC1 resulted in an *r*^2^ of 0.8 (**F**). White dots indicate the colocalization of mMCT1 and mPC1 as observed in ImageJ software. Whole images were contrast-enhanced in (**A**, **B**, **C**, **D**, and **E**). Similar results were observed for mPC2. Scale bar = 20 µm. It appears that both MCT1 and the putative mPC colocalized to the mitochondria (*r*^2^ = 0.8). However, at the light microscopic level, it is impossible to know if the two proteins interact physically and functionally. Additionally, with the benefit of the Orbitrap LC/MS device, we could determine the fractional synthesis rates of mLOC and mPC proteins. From Brooks^6^.

Analogous to the controversial history of mLDH discovery was the discovery of the presence of mitochondrial monocarboxylate transporters (mMCTs)^13,50,68^. These were visualized in adult rat skeletal muscle^154^ and cultured L6 myocytes by Takeshi Hashimoto and colleagues^153^, Figs. 3 and 4, respectively. For a time, the isoform monocarboxylate transporter 1 (i.e., MCT1) was the only lactate/pyruvate transporter known to be expressed in the mammalian mitochondrial proteome^57^. Subsequently, the mitochondrial pyruvate transporter (mPC) was identified^160,161^. Unfortunately, at present, little effort has been expended to evaluate functional or morphometric relationships between mPCs and mMCTs, particularly in mammalian skeletal muscle. Indeed, we cannot find reports in the literature of an mPC in mammalian or human skeletal muscle. Reports of an mPC in yeast^160,161^ are unconvincing because of single-band Western blots that are unaccompanied by a ladder to assess the molecular weights of the putative mPC. However, with access to our own custom antibodies to MCT1, as well as a commercially available antibody to the putative mPC, we obtained images assessing the colocalization of MCT1 and mPCs in L6 cells. In those preliminary studies, colocalization analysis of mMCT1 and mPC1 in Imaris software showed an *r*^2^ of 0.8. It appears that both MCT1 and mPCs are colocalized to the mitochondria (*r*^2^ = 0.8) (Fig. 7)^6^. However, at the light microscopic level, it is impossible to know whether mMCTs and mPCs interact physically and functionally. Immunoprecipitation, X-ray crystallography, mass spectrometry, and protein deletion (knockout) studies are needed to definitively answer questions about mMCT and mPC colocalization and functionality and the role of mPCs in mitochondrial lactate oxidation.

### Role of the mitochondrial lactate oxidation complex (mLOC) in lactate/pyruvate metabolism

Discovery of the mLOC (Fig. 5) and its role in Cell–Cell and Intracellular lactate shuttles may cause some traditional thinkers to assert something such as “the diversion of glycolytic flux from pyruvate to lactate via cytosolic LDH (cLDH) with a return to pyruvate via mLDH still means that pyruvate is the major mitochondrial energy substrate”. Such an assertion would hold at the unicellular organismic level. However, to briefly reiterate from above, lactate shuttles are based on lactate concentration differences between producer and recipient cells connected through the interstitium within a tissue bed or the vasculature at the organ system level. In the shuttling of carbohydrate carbon energy substrates, lactate concentrations are orders of magnitude greater than those of pyruvate and alanine. Hence, corporal energy substrate distribution is accomplished with lactate, not pyruvate, as the vehicle of mass-energy transfer^60^.

### The mitochondrial lactate oxidation complex (mLOC) in cancer

As noted above, in the Brooks laboratory, Takeshi Hashimoto et al. showed the presence of the mLOC in mammalian skeletal muscle, muscle cell lines, neurons, and primary neuronal cell cultures^138,153,154^. In addition, his work revealed the presence of a lactate-sensitive transcription network in cultured myocytes^155^. Having been part of the team previously working on muscle and brain, both postmitotic tissues, Rajaa Hussien in the Brooks laboratory commenced work on breast cancer cells^140^ that display active mitosis. Initial efforts were on known mLOC proteins, including monocarboxylate transporters (MCT1, MCT2, and MCT4), a scaffold glycoprotein (CD147), lactate dehydrogenase (LDH isoforms A and B), and cytochrome oxidase (COx). The results were consistent with their primary hypothesis that mLOC expression and assembly are dysregulated in breast cancer^140^. Hussien and Brooks tested their primary hypothesis on the breast cell lines MCF-7, MDA-MB-231 and HMEC-184; the former two are transformed breast cell lines that are cancerous, while HMEC-184 is a nontransformed primary breast cell line that was used as a control. Protein expression of MCTs, CD147 and LDHs was determined by immunoblot analysis of whole-cell extracts from the three cell lines grown under standard conditions. The expression of the seven proteins in the two transformed cell lines was compared with that in the primary untransformed cell line HMEC-184. Relative to HMEC-184, in the transformed cells, some proteins (MCT2, LDH and CD147) were upregulated. Remarkably, the expression of MCT1 was low in MCF-7 cells and undetectable in MDA-MB-231 cells. A notable novel discovery from their study was the reciprocal relationship between the MCT1 and CD147 protein levels. The data from breast cancer cell lines led to the hypothesis (as yet incompletely explored) that the dysregulation of the coordinated expression of those two proteins and their respective genes contributes to the carcinogenic process in breast cancer.

**Fig. 8:**
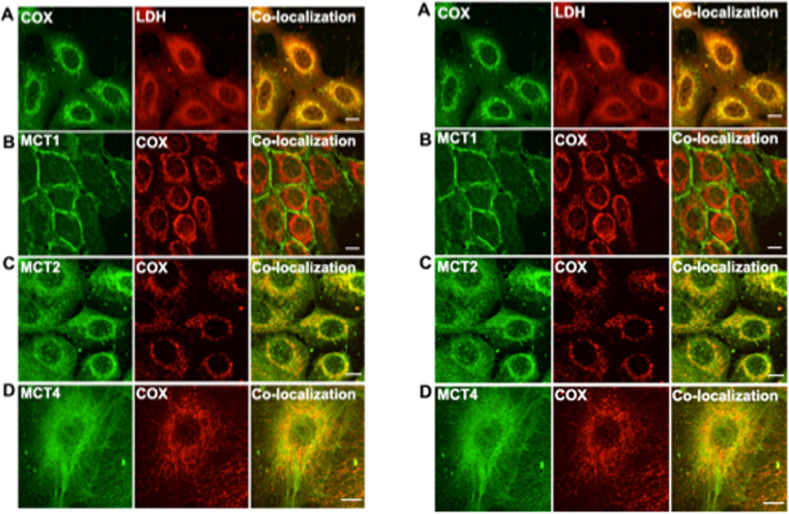
Differential cellular localizations in mitochondrial Lactate Oxidation Complexes (mLOC) proteins in normal and cancer breast cell lines. Immunohistochemical detection of LDH, MCT isoforms, and cytochrome oxidase (COx) in the control breast cell line HMEC-184, Plate **A**, left, and in the breast cancer cell line MCF-7, Plate **B**, right. LDH isoforms MCT2 and MCT4 colocalized with the mitochondrial protein marker COx (rows **A**, **C**, **D**) but not MCT1, which was localized mainly in the plasma membrane in the control HMEC cell line (Plate **B**, Row B, left). Thus, mMCT isoform expression in breast cell mitochondria differs from that in skeletal muscle, where MCT1 predominates (Figs. 4 and 5); in control and cancer breast cells, MCT-2 and -4 isoforms colocalized with the inner mitochondrial membrane component COx. The thickness of the optical sections, ~1 µm, scale bar = 1 µm. Images from Hussien and Brooks^140^.

To probe for the presence of mLOC proteins in cancer cells, Hussien and Brooks^140^ also examined the subcellular localization of MCTs and LDH isoforms in the three cell lines studied. Confocal laser scanning microscopy and related immunohistochemical techniques showed the presence of MCTs, LDH isoforms, and COx. LDH isoforms MCT2 and MCT4 were colocalized with the mitochondrial protein marker COx, but distinct from the situation in muscle, MCT1 was not mitochondrial and was localized exclusively in the plasma membrane. The localization of MCT and LDH isoforms in the two cancer cell lines (MCF-7 and MDA-MB-231) and the normal cell line (HMEC-184) was the same. MCT2, MCT4, and LDH were localized in mitochondria in addition to their localization in the plasma membrane and cytosol, whereas MCT1 was mainly localized in the plasma membrane (Fig. 8). Their data showed that in a breast cancer cell line, changes in the expression of lactate shuttle proteins occur. The reasons for these changes in mLOC protein expression, transcription, translation, and turnover in breast cancer need elucidation.

## Lactate signaling

The effects of increases in cellular work and subsequent lactate production on redox, reactive oxygen species (ROS), allosteric binding, and histone lactylation have been recently reviewed^5^. Briefly, lactate affects metabolic signaling via diverse mechanisms that include changing the L/P and NADH/NAD^+^ and thus cell redox; activating sirtuins via the enzyme NAM phosphoribosyl transferase (Nampt); stimulating transforming growth factor beta 2 (TGF-β2) secretion from adipose tissue; regulating gene expression by the lactylation of 28 lysine residues on histones^162^; and inhibiting lipolysis by activating Hydroxycarboxylic Acid Receptor-1 (HCAR-1) operating through cyclic AMP (cAMP) and cAMP response element-binding (CREB^163,164^. With regard to gene regulation via histone lactylation, it should be noted that it was discovered using tracer methodology ([U-^13^C]lactate and [U-^13^C]glucose) and a cancer cell line (MCF-7). As noted previously, the purported effects of lactate signaling via HCAR-1, TGF-β2, Nampt, and the lactylation of histones observed in rodent muscle and brain and cell line models await validation in humans^4^. The latter statement is appropriate because of the growing interest in evaluating the role of lactylation in promoting carcinogenesis in a variety of cell types, including those of the lungs^165^. Regrettably, thinking in the field is limited by reliance on outmoded O_2_ debt (i.e., O_2_-limited) ideas of metabolic regulation^166^.

## Lactate and mitochondrial biogenesis

Endurance exercise has long been recognized to stimulate mitochondrial biogenesis^167,168^. Hence, the following question arose: ‘Could the lactatemia of exercise stimulate mitochondrial biogenesis?’ In an attempt to address the question, Hashimoto and colleagues incubated L6 cells in elevated sodium lactate added to buffered, high-glucose media and screened the cells’ genome-wide responses^155^. Lactate increased ROS production and upregulated 673 genes, many known to be responsive to exercise and exercise training via ROS and Ca^++^ stimulation. The induction of genes encoding components of the mLOC was confirmed by a polymerase chain reaction and electrophoretic mobility shift assay (PCR and EMSA, respectively). Among the many effects, lactate increased the mRNA and protein expression of MCT1 and COx. Increases in COx expression coincided with increases in peroxisome proliferator activated-receptor gamma coactivator-1 alpha (PGC1α) expression and the DNA binding activity of nuclear respiratory factor (NRF)-2. Based upon their results, Hashimoto et al. concluded that the presence of a lactate signaling cascade involves ROS production and converges on transcription factors affecting mitochondrial biogenesis.

The presence of a lactate-sensitive transcription factor network and its putative effects on muscle mitochondrial biogenesis in exercising humans have yet to be evaluated. To date, Genders et al.^169^ in the David Bishop Laboratory have confirmed elements of the work of Hashimoto et al.; specifically, they have advanced the field to show that low or high pH can suppress the effect of lactate on histone deacetylase (HDAC) activity and protein kinase B (aka, Ak strain transforming, Akt) signaling in L6 cells. Studies on the possibility of exercise above the lactate threshold, where there is a disproportionate increase in muscle lactate production, activating a signaling cascade leading to human skeletal muscle mitochondrial biogenesis are underway (David Bishop personal communication).

## The lactate story comes full circle

Considering the above text on intact individuals in vivo, tissues, and organs in situ, and cells in vitro, as well as the results on cancer cells to date, it seems that the study of lactate metabolism has come full circle. From the perception of a lowly waste product formed as the result of a lack of oxygen, as embodied in the shuttle hypothesis, lactate is now realized to have three essential metabolic functions: an energy source, a gluconeogenic precursor, and a signaling molecule. Originally conceived of within the context of exercise physiology and supporting short- and long-term adaptive processes, some investigators propose using lactate esters and salts to sustain athletes in prolonged hard exercise bouts^170^ and to provide nutritive support to humans following traumatic brain injury^104^.

Regrettably, in clinical settings, the understanding and use of lactate in diagnosis and treatment is inadequately understood and employed. For instance, half molar lactate infusion is useful to supply nutritive support to patients with heart failure and following myocardial infarction^171^. Similarly, in the realm of traumatic brain injury^104^, a clinical trial is underway to evaluate the efficacy of lactate supplementation to support the injured brain. Nevertheless, as lamented by See and Bellomo^172^, in intensive care units where the blood lactate level is a biomarker for the severity of sepsis, a lack of knowledge on the sites and reasons for lactatemia hampers treatment. Fortunately, progress has been made in understanding the role of lactatemia in the etiology of diabetic ketoacidosis^173^. Similarly, the role of dysregulated lactate metabolism is advancing, with investigators giving thought to destroy cancer cells by blocking lactate shuttling^140,141,151,174,175^.

## Summary

A chronology of significant events and important discoveries in the field of lactate metabolism is given in Table 1. As shown, in the latter part of the 20th century, the use of isotope tracer technologies enabled significant contributions to a revolution in understanding the diverse roles of lactate in the regulation and integration of intermediary metabolism in humans and other mammals in health and disease.

**Table 1:** Historical timeline of lactate metabolism thought and discoveries.

**1907–1924**: The Lactic Acid Era. Archibald Vivian (A.V.) Hill and the Cambridge School of Physiologists believed that the “processes of muscle contraction are due to the liberation of lactic acid from some precursor”^22–24,176^. Otto Meyerhof quantifies the relationship between glycogen and lactic acid formation in isolated, nonperfused frog hemicorpus preparations^25–27^. A.V. Hill develops the O_2_ Debt concept in human studies^65–67,177^. August Krogh and Johannes Lindhard^178^ provide evidence supporting Zuntz’s assertion that both fat and carbohydrate are substrates for energy during exercise in humans^179^, but those studies were outside the prevailing theory of the Cambridge School of A.V. Hill and associates.
**1925**: Contrary to the oxygen-limited, O_2_ Debt Ideas of the Cambridge school, contemporaneously in Germany, Otto Warburg describes aerobic glycolysis in tumor cells; subsequently the phenomenon became known as the “Warburg Effect”^64,180^. Later, it became obvious that lactate production occurs in the soma of healthy individuals while resting or exercising or after carbohydrate nutrition^181^ as well as in the gut microbiome^3^.
**1929:** Carl Ferdinand Cori and Gerty Theresa Cori propose and describe a metabolic pathway in which L-lactate produced by anaerobic glycolysis in the muscles moves to the liver where it is oxidized to pyruvate to glucose, which then returns to the muscles and is metabolized^182^. This was the first expression of a Lactate Shuttle mechanism involving the vascular exchange of precursor from a driver cell or tissue (muscle) and receipt by a recipient cell or tissue (liver), and return.
**1933**: Rodolfo Margaria, Harold T. Edwards and David Bruce Dill of the Harvard Fatigue Laboratory apply the new knowledge of the phosphagens to O_2_ Debt theory in humans and segment the phenomenon into “lactacid” and “alactacid” components^183^.
**1936:** Ole Bang challenged the established lactacid O_2_ debt concepts^184^. But, his results are ignored, WW II intervenes and oxygen debt ideas became entrenched.
**1937, 1938** Data of Hans Krebs and William Johnson^185^ and Krebs et al.^186^ indicate that the carbohydrate derivative to enter the Tricarboxylic Acid Cycle (TCA) is pyruvate. The role of mitochondrial lactate dehydrogenase (mLDH) is unknown and unsuspected.
**1940:** After the elucidation of the glycolytic pathway (known as the Embden–Meyerhof–Parnas Pathway) that assumed pyruvate to be its end product under aerobic conditions, together with conclusions of Krebs and associates that pyruvate is the molecule that enters the TCA, most of the research into energy metabolism would continue unabated using the basic paradigm established by the principal players of the first half of the 20th century.
**1951:** Mario Umberto Dianzani shows that rat liver mitochondria oxidize lactate^78^.
**1955 & 1956**: Douglas S. Drury, Arne N. Wick and Toshiko Morita use ^14^C-lactate tracers to show extra-hepatic oxidative disposal of lactate in rabbits^97,187^.
**1964**: Karlman Wasserman and Malcolm B. McIlroy assume the Meyerhof-Hill O_2_-Debt theory of oxygen-limited lactate production and coin the term “anaerobic threshold”, which purported to describe the “onset of anaerobic metabolism during exercise”^188^.
**1966–1968**: Wendell Stainsby and Hugh Welch demonstrate the transient nature of muscle lactic acid output in canine muscle in situ and present evidence that argues for the O_2_ independence of lactic acid formation during muscle contractions^105,106^. This phenomenon is to be observed in exercising humans; in 1998, Brooks and colleagues observe the same phenomenon in humans and name it the “Stainsby Effect”^189^.
**1968** Franz F. Jöbsis and Wendell Stainsby demonstrate oxidation of Complex 1 in the mitochondrial electron transport chain of dog gastrocnemius muscles contracting in situ at an intensity sufficient to produce and release lactate^190^.
**1969**: Florent Depocas and colleagues use radioactive tracers to study lactate turnover and oxidation in resting and exercising dogs. Fundamental discoveries of Depocas et al. showing active lactate turnover in resting and exercising individuals have been replicated on numerous species, including humans^69^.
**1970**: Albert Lehninger publishes the textbook Biochemistry^41^. Concepts of glycolysis and fermentation are confused. Ideas that glycolysis leading to pyruvate under aerobic conditions and that lactate is produced due to oxygen lack were enshrined in the latter part of the 20th century. Only recently has a contemporary understanding of distinctions between fermentation and glycolysis, and the role of oxygen, or rather the lack thereof, in determining the end product of glycolysis which is lactate have started to emerge in contemporary basic biology textbooks ^40^.
**1971**: Nobuhisa Baba and Hari M. Sharma visualize mitochondrial LDH using electron microscopy. They were the first to postulate a “Lactate Shuttle”, but did not follow up on their observation^53^.
**1973**: George A. Brooks and associates give ^14^C-lactate to rats after exhausting exercise and find, contrary to classic “O_2_ Debt” theory, little incorporation of lactate into glycogen but major disposal as ^14^CO_2_. This is the first challenge to the classic Hill-Meyerhof concept of a 1/5 – 4/5 lactate-to-lactate conversion to glycogen ratio, which is converse of what happens in a mammalian system after exercise^191^.
**1980**: Glenn A. Gaesser and George A. Brooks use bolus injections of [U-^14^C]glucose and -lactate tracers, indirect calorimetry, and two-dimensional chromatography to trace the paths of lactate and glucose disposal during recovery from exhausting exercise. Again, they find little incorporation of lactate-derived carbon into glycogen but major disposal as ^14^CO_2_. In mammals, oxidation, not reconversion to glycogen, is the major fate of lactate after exercise^116,117^.
**1983**: Casey M. Donovan and George A. Brooks use primed continuous infusions of [U-^14^C]glucose and -lactate tracers, indirect calorimetry, and two-dimensional chromatography to determine the flux, oxidation, and conversion rates of lactate to glucose in endurance-trained and untrained rats during exercise. Training results in the classic finding of lowered arterial [lactate] that is due to increased clearance via oxidation and gluconeogenesis^71,124^. This seminal study on lab rats and the resulting paper was subsequently reproduced using stable, nonradioactive tracers on human subjects^35,112–114,192,193^.
**1984**: Richard Connett, Tom Gayeski, and Carl Honig observe lactate production in canine muscle in situ when intramuscular PO_2_ is apparently above the critical value for mitochondrial oxidative phosphorylation^30^.
**1984**: Lactate Shuttle Hypothesis Articulated: Based on tracer-measured glucose and lactate fluxes and biochemical evidence from the Kenneth M. Baldwin Lab^194,195^, George Brooks proposes the Lactate Shuttle in a meeting of comparative physiology in Liege, Belgium. Meeting proceedings are published the following year^125^.
**1984**: Daniel Foster presents at the annual Banting Lecture, revealing the importance of lactate to hepatic glycogen synthesis (the ‘Indirect Pathway’) following carbohydrate nutrition^196^. In retrospect, Foster’s work anticipated the Postprandial Lactate Shuttle^181^.
**1984**: George A. Brooks and Thomas D. Fahey publish the first edition of EXERCISE PHYSIOLOGY: HUMAN BIOENERGETICS AND ITS APPLICATIONS^118^. Contemporary ideas of lactate metabolism appear in a textbook. In two volumes, the work is now in its fifth edition^46,197^. Textbook versions of the Oxygen Debt and Anaerobic Threshold were fundamentally changed.
**1988**: Peter W. Watt and Peter A. MacLennan^134^ describe the characteristics of muscle lactate exchange kinetics, pH dependence, and competitive inhibition and saturation of lactate exchange in perfused rat skeletal muscle preparations.
**1988:** For the first time, Avital Schurr and colleagues described the ability of lactate to support synaptic function of hippocampal neurons in vitro as the sole source of energy substrate^198^.
**1990**: David Roth and George Brooks describe the characteristics of sarcolemmal lactic acid transport^132,133^. Concentration and pH dependence, competitive inhibition, and saturation kinetics were demonstrated, as predicted from Watt and MacLennan^134^.
**1990:** Szczesna-Kaczmarek demonstrates L-Lactate oxidation by skeletal muscle mitochondria^19^.
**1991**: George Brooks proposes the Intracellular Lactate Shuttle based on measured muscle lactate exchange and oxidation in vivo^199^.
1**992**: Szczesna-Kaczmarek demonstrates control of mitochondrial L-Lactate oxidation by LDH^19^.
**1994**: Christine Kim Garcia, Michael S. Brown, Joseph L. Goldstein, and colleagues sequence and clone the gene encoding for a muscle cell membrane monocarboxylate transport protein (MCT)^135^.
**1995**: Garcia and colleagues identify a second isoform, MCT2, located mainly in the liver^137^.
**1997:** Avital Schurr and associates demonstrated that brain lactate, not glucose, fuels the recovery of synaptic function from hypoxia upon reoxygenation in vitro^200^.
1**998**: Russ Richardson, Peter Wagner, and colleagues use magnetic resonance spectroscopy (MRS) to show lactate production and net release from fully aerobic, working human skeletal muscle^32^.
**1998**: Andrew Halestrap and colleagues clone and sequence four new MCT isoforms and describe tissue variability in MCT isoform expression^142^.
**1998**: An Astrocyte-Neuron Lactate Shuttle was proposed by Pierre Magistretti, Luc Pellerin^139,201^, and colleagues.
**1999**: Paul Molé and colleagues^31^ use MRS to confirm results of Richardson et al.^32^ showing lactate production and net release from fully aerobic, working human skeletal muscle.
**1999**: Henriette Pilegaard, Andrew Halestrap and Carsten Juel show MCT1 and MCT4 distribution in human skeletal muscle^202,203^.
**1999**: Brooks, Marcy Brown, Hervé Dubouchaud, and colleagues show LDH and MCT1 in muscle mitochondria of rats^13,50^.
**2000**: Hervé Dubouchaud and colleagues in the Brooks Lab confirm the presence of mLDH and mMCT1 in human skeletal muscle mitochondria^15^.
**1999 & 2000**: Bryan Bergman, Eugene Wolfel, Gail Butterfield, Gretchen Casazza, Michael Horning, Hervé Dubouchaud, George Brooks, and colleagues show that endurance training improves lactate clearance by intramuscular oxidation and gluconeogenesis in humans^35,113,193,204^. These results confirm and extend the 1983 studies on lab rats^71,124^.
**2001**: Avital Schurr et al. showed that blockade of lactate transport via MCT1 exacerbates neuronal damage in a rat model of cerebral ischemia^205^.
**2002**: Recognition of lactate as a signaling molecule, a lactormone”^89^.
**2002**: Benjamin Miller, George Brooks, and colleagues use exogenous lactate infusion (“Lactate Clamp”) and stable isotope tracer technology to test lactate clearance mechanisms and show preferential lactate over glucose oxidation in exercising men^206,207^.
**2002**: Daniela Valenti, Lidia De Bari, Anna Atlante*, and Salvatore Passarella publish “L-Lactate transport into rat heart mitochondria and reconstruction of the L-lactate/pyruvate shuttle”^208^.
**2003:** Anne Karine Bouzier-Sore et al. showed lactate to be the preferential energy substrate over glucose for neurons in culture^209^.
**2003:** Diarmuid Smith and colleagues demonstrate that lactate is a preferred fuel for human brain metabolism in vivo^210^.
**2004**: Robert A. Robergs and colleagues illustrate that lactate anions, not lactic acid (pKa = 3.86), are formed during exercise.
**2004**: C. Eric Butz and Grant McClelland reconfirm the presence of mLDH and mMCT1 in skeletal muscle mitochondria^68^.
**2006**: Takeshi Hashimoto and George A. Brooks and colleagues provide evidence of a mitochondrial lactate oxidation Complex (mLOC) comprised minimally of mLDH, mMCT1, basigin (CD147), and cytochrome oxidase (COx) in rodent skeletal muscle^153^.
**2006**: Avital Schurr hypothesizes that lactate is the ultimate cerebral oxidative energy substrate^147^.
**2007**: Takeshi Hashimoto and George Brooks and colleagues provide evidence of gene regulation by lactate. In L6 (rat muscle-derived) cells, the upregulated genes include those encoding for MCT1, mitochondrial proteins, proteins involved in ROS generation and quenching, and calcium response elements^155^.
**2007**: Schurr and Payne show that lactate, not pyruvate, is the end product of glycolysis in neurons under aerobic conditions^150^.
**2007**: John T. (Jack) Azevedo and colleagues demonstrate preferential lactate oxidation over glucose and fructose when co-ingested during continuous exercise^170^.
**2008**: Takeshi Hashimoto and George Brooks and colleagues provide evidence of a mitochondrial lactate oxidation Complex (mLOC) comprised minimally of mLDH, mMCT1, basigin (CD147), and cytochrome oxidase (COx) in rat neurons^138^.
**2008**: Pierre Sonveaux and colleagues find MCTs and cell–cell lactate shuttling to be prevalent among tumor cells. They used siRNAs and MCT pharmacological to kill cancer cells in vitro and in mice in vivo, thus making MCTs targets for cancer treatment^141^.
**2009**: Garret van Hall, Niels Secher, and colleagues show cerebral lactate uptake and oxidation in exercising humans^84^.
**2010**: Kashan Ahmed and colleagues elucidate a lactate-mediated autocrine response through an orphan G protein (GPR81), now known as hydroxycarboxylic acid receptor-1 (HCAR-1), demonstrating the ability of lactate to downregulate levels cAMP levels and therein having an antilipolytic effect^163^.
**2010**: Lidia de Bari, Daniela Valenti, Anna Atlante, and Salvatore Passarella provide evidence of the presence of an intermembrane lactate oxidase that generates H_2_O_2_ sufficient to activate the known ROS response elements signaling mitochondrial adaptation and other adaptations to exercise. This work provides a mechanism by which lactate generation in muscle exercise participates in the feedback loop by which lactate generation in exercise leads to adaptations facilitating high rates of lactate disposal in exercise^14^.
**2011**: Rajaa Hussien and George Brooks find differences in mitochondrial LDH and MCT isoform expression in normal breast cancer and breast cancer cells^140^.
**2011**: Avital Schurr publishes “Lactate: the ultimate cerebral oxidative energy substrate?”^147^.
**2011:** Schurr and Gozal showed that aerobic production and utilization of lactate satisfy increased energy demands upon neuronal activation in hippocampal slices and provide neuroprotection against oxidative stress^149^.
**2011**: Luc Pellerin and Pierre Magistretti celebrate the 16^th^ year of proposing the Astrocyte-Neuron Lactate Shuttle (ANLS). Neglected was that the ANLS was proposed 14 years after the seminal lactate shuttle papers^126,127^.
**2013**: Robert A. Jacobs and colleagues reconfirm mitochondrial lactate oxidation in mitochondrial preparations from human skeletal muscle ^56^.
**2014**: Salvatore Passarella and colleagues publish “The mitochondrial L-lactate dehydrogenase affair“^7^.
**2014**: Avital Schurr publishes “Cerebral glycolysis: a century of persistent misunderstanding and misconception”^8^.
**2016–2021**: Results of studies showing lactate shuttling in rabbits, dogs, horses, and humans confirmed in mice^211–214^.
**2019**: By the addition of exogenous lactate and U^13^-C_6_-Glucose, Di Zhang and colleagues identify 28 lactylation targets on core histones, illustrating another epigenetic modification by which the genome is regulated^162^. Knowledge of the role of the role of lactate as a signal is extended^3,5,89,155,215^.
**2020**: Adrian Young and colleagues reconfirm mitochondrial LDH and lactate oxidation in mitochondrial preparations from mouse liver, cardiac, and skeletal muscle, yet again^54^.
**2020:** David C. Poole, Harry B. Rossiter, L. Bruce Gladden, and George A. Brooks review 50 years of controversy on the anaerobic threshold and interpret results in terms of Lactate Shuttle Theory^216^.
**2021** Brooks and graduate students articulate the Postprandial Lactate Shuttle^181^.
**2021:** Brooks and graduate students review the role of the heart in lactate shuttling^102^.
**2021:** Brooks and graduate students explain, and review false claims about use of lactate tracers to study metabolism^60^.

*Updated from ref. ^189^ and inspired by Avital Schurr and Salvatore Passarella.

Isotope tracer technologies were central to the deduction and demonstration of the Lactate Shuttle at the whole-body level. In concert with the ability to perform tissue metabolite concentration measurements, as well as determinations of unidirectional and net metabolite exchanges by means of a-v difference and blood flow measurements across tissue beds including muscle and the brain, lactate shuttling within organs and tissues was evident. We now know that Organ–Organ, Cell–Cell, and Intracellular Lactate Shuttles operate continuously, even after eating when a Postprandial Lactate Shuttle is evident^4^. By means of lactate shuttling, fuel-energy substrate exchange occurs between tissues and organs such as muscle and the brain; muscle and the heart; muscle and the liver, kidneys, and gut; and muscle and other tissues. A muscle-like role of the integument is also highly suspected but unexplored. A Corporal-Microbiome Lactate Shuttle has been proposed^3^ but is also unexplored. Within tissues, lactate can be exchanged between white and red fibers within a muscle bed and between astrocytes and neurons in the brain. Within cells, lactate can be exchanged between the cytosol and mitochondria and between the cytosol and peroxisomes. By means of isotope tracers and related methodologies, we know that there are at least three general functions of lactate shuttling: lactate is an energy source preferred over glucose in the heart, red skeletal muscle, and brain; lactate is the major gluconeogenic precursor; and lactate is a signaling molecule. Lactate signals otherwise affect energy substrate partitioning by mass action, cell redox, the lactylation of histones, and covalent binding to HCAR-1 and signaling via CREB-dependent pathways. Discovered in the realm of exercise physiology and biochemistry, it is now possible to recognize a Postprandial Lactate Shuttle by which dietary carbohydrates are absorbed, circulated, and taken up and used or stored in diverse tissues. Isotope tracers and related technologies have made multiple fundamental discoveries possible in the field of metabolic regulation.
